# TRAIL/S-layer/graphene quantum dot nanohybrid enhanced stability and anticancer activity of TRAIL on colon cancer cells

**DOI:** 10.1038/s41598-022-09660-5

**Published:** 2022-04-07

**Authors:** Shima Lotfollahzadeh, Elaheh Sadat Hosseini, Hooman Mahmoudi Aznaveh, Maryam Nikkhah, Saman Hosseinkhani

**Affiliations:** 1 Department of Nanobiotechnology, Faculty of Biological Sciences, Tarbiat Modares University, P. O. Box: 14115-154, Tehran, Iran; 2 Department of Biochemistry, Faculty of Biological Sciences, Tarbiat Modares University, P. O. Box: 14115-154, Tehran, Iran

**Keywords:** Diagnostics, Drug delivery, Cancer imaging, Cancer therapy

## Abstract

Tumor necrosis factor (TNF)-related apoptosis inducing ligand (TRAIL), known as a cytokine of the TNF superfamily, is considered a promising antitumor agent due to its ability to selectively induce apoptosis in a wide variety of cancer cells. However, failure of its successful translation into clinic has led to development of nano-based platforms aiming to improve TRAIL therapeutic efficacy. In this regard, we fabricated a novel TRAIL-S-layer fusion protein (S-TRAIL) conjugated with graphene quantum dots (GQDs) to benefit both the self-assembly of S-layer proteins, which leads to elevated TRAIL functional stability, and unique optical properties of GQDs. Noncovalent conjugation of biocompatible GQDs and soluble fusion protein was verified via UV–visible and fluorescence spectroscopy, size and ζ-potential measurements and transmission electron microscopy. The potential anticancer efficacy of the nanohybrid system on intrinsically resistant cells to TRAIL (HT-29 human colon carcinoma cells) was investigated by MTT assay and flow cytometry, which indicated about 80% apoptosis in cancer cells. These results highlight the potential of TRAIL as a therapeutic protein that can be extensively improved by taking advantage of nanotechnology and introduce S-TRAIL/GQD complex as a promising nanohybrid system in cancer treatment.

## Introduction

Despite remarkable achievements in cancer treatment by limiting cancer cell proliferation or inducing programmed cell death, cancer remains a major challenge. Current interests have concentrated on cancer cell-specific apoptosis, which has long been a desire for targeted cancer therapy^1–3^.

Tumor necrosis factor (TNF)-related apoptosis inducing ligand (TRAIL/Apo2 L), known as a cytokine of the TNF superfamily, is a type II transmembrane protein ligand that induces caspase-dependent apoptosis via the extrinsic pathway in a wide variety of human cancer cells while having negligible cytotoxicity toward normal cells^4–6^^.^ This homotrimeric ligand binds to two proapoptotic death receptors known as TRAIL-R1 (DR4) and TRAIL-R2 (DR5), which are highly expressed in malignant cells, unlike healthy cells^7–9^.

Regardless of the potential of TRAIL as a promising cancer therapeutic agent, soluble human TRAIL clinical applications have been hampered due to its weak pharmacokinetic profile, extremely poor circulation and short serum half-life, rapid renal elimination, insufficient delivery to the target site and TRAIL resistance in some cancer cells^10–12^. The inability of the soluble form of TRAIL to assemble as a homotrimeric structure could be another factor involved in this failure^13,14^. To date, intense research efforts have been made to bring these formidable hindrances under control and enhance TRAIL stability and activity^10,15–17^.

One approach that has been extensively utilized in prior studies is fusing TRAIL to other proteins or using a variety of nanobased drug delivery systems, which have represented a promising step forward^6,10,16,17^. Here, with the aim of TRAIL stability and bioactivity improvement, TRAIL was fused to crystalline bacterial cell surface layer (S-layer) protein. S-layers, which are two-dimensional arrays of protein or glycoprotein subunits, have been identified as the outermost envelope structure of archaea and in cell-surface components of approximately all walled bacteria^18,19^. It has been shown that isolated native s-layer proteins or their fusion proteins self-assemble in defined orientations into arrays in suspension or recrystallize at the liquid-surface interfaces and on solid supports^19–21^.

**Figure 1:**
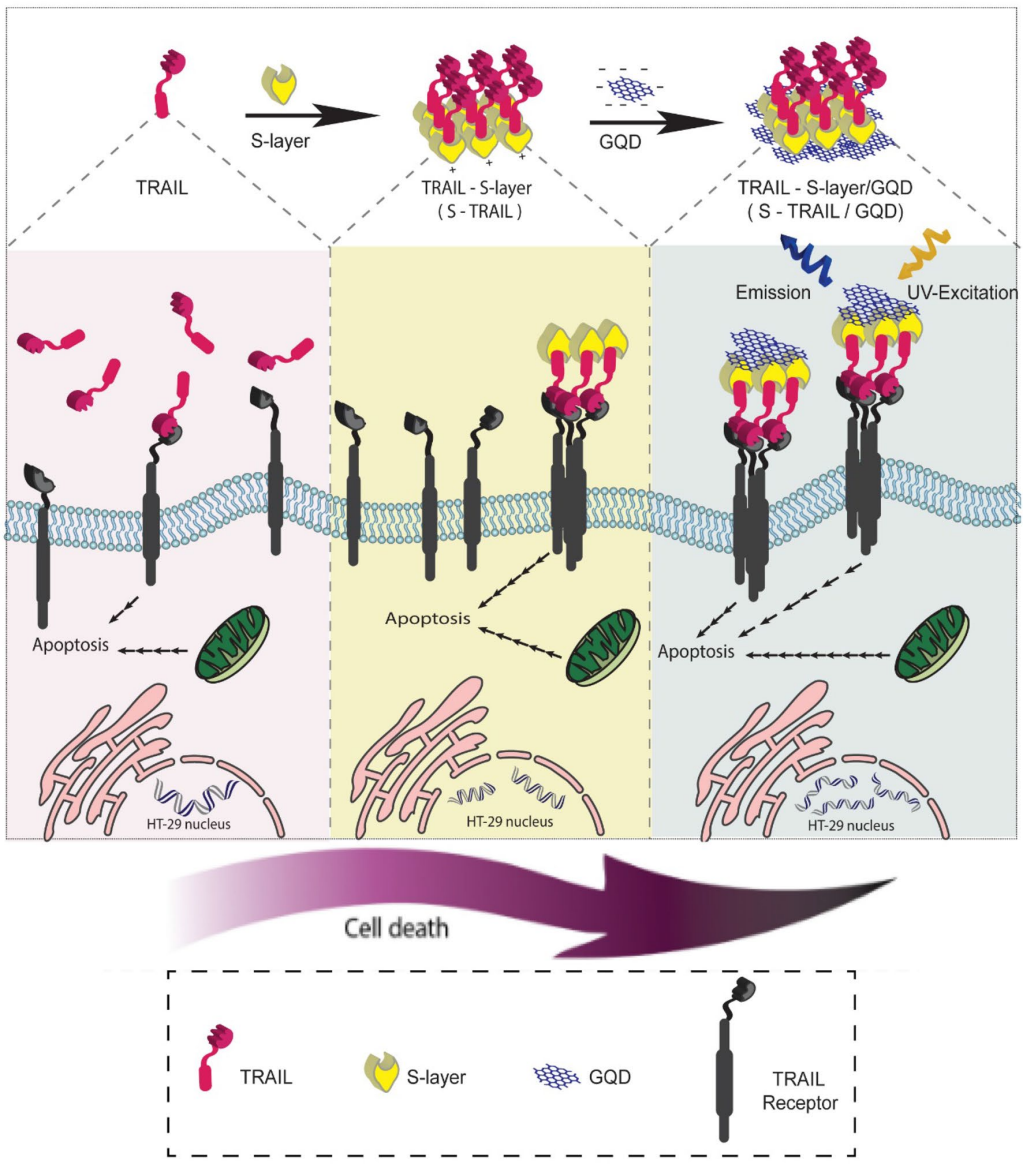
Schematic illustration of S-TRAIL and S-TRAIL/GQD preparation and biological function.

Here, a novel TRAIL-S-layer fusion protein (S-TRAIL) was conjugated to graphene quantum dots (GQDs) through noncovalent interactions (Fig. 1). In the designed nanohybrid system, the self-assembly of the S-layer protein is expected to improve the biological and physicochemical stability and biological function of TRAIL. The GQDs provide both a biocompatible surface for self-assembly of the fusion proteins and enable the tracking of the cargo. The N-terminus of the S-layer protein of Lactobacillus acidophilus ATCC 4356 bacteria (slpA), which is required for its self-assembly, was linked to TRAIL by a G4S linker. The C-terminal domain of the S-layer protein, which is responsible for its binding to the cell wall, was expected to bind to negatively charged GQDs through electrostatic interactions^22,23^.

The physicochemical characteristics of S-TRAIL/GQDs and their stability were evaluated. The cytotoxicity of the nanohybrid system on HT-29 human colon carcinoma cell line was assessed by MTT assay and flowcytometry. The obtained results indicated that S-TRAIL/GQD could be introduced as a promising theranostic candidate for cancer therapy.

## Results and discussion

### TRAIL, S-layer and S-TRAIL expression and purification

**Figure 2:**
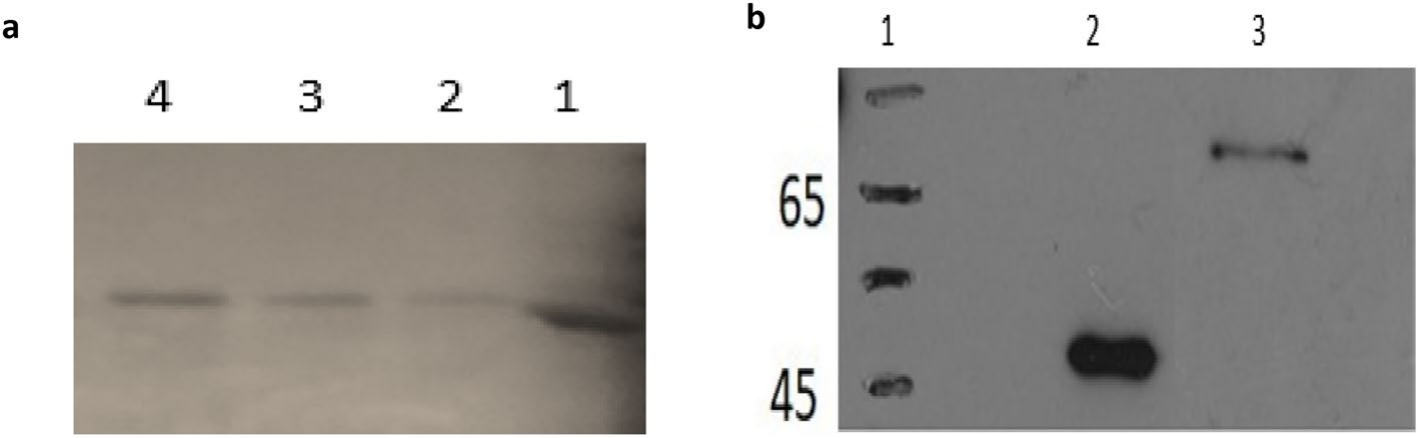
(**a**) Analysis of purified S-TRAIL using Comassie blue stained SDS-PAGE (12.5% polyacrylamide gel). Lane1. Molecular weight marker (62KDa), Lane 2–4. S-TRAIL (MW ~ 66 KDa). (**b**) Western blot analysis of S-layer and S-TRAIL using monoclonal anti-His tag antibody and goat anti-mouse AP conjugated antibody. Lane1. Molecular weight marker, Lane2. S-layer and Lane3. S-TRAIL.

Expression of S-TRAIL recombinant protein in BL-21(DE3) *E.coli* and its purification by one step Ni-affinity chromatography, after multistep optimization in terms of IPTG concentration, induction time and temperature, led to a high level of expressed soluble S-TRAIL with final concentration of 8 mg/l of bacterial culture. A single band with molecular size of approximately 66 KDa was observed in both SDS-PAGE (Fig. 2a) and western blot (Fig. 2b) analyses.

One approach that has been extensively utilized in prior studies is fusing TRAIL with different tags or other proteins to facilitate its purification and trimerization and also to enhance its stability and bioactivity, which consequently leads to improvement of its therapeutic efficacy. Wiley et al. in 1995 designed the first recombinant version of TRAIL with an added FLAG epitope tag^24^. This approach was followed by adding other tags, motifs and domains to the extracellular domain of TRAIL^25–27^, fabricating various fusion proteins^28–31^, or by trying to set up innovative approaches to modify TRAIL purification^32–35^. However, despite all valuable progress in improving bioactivity, TRAIL purification still suffers from aggregation and formation of inclusion bodies, requiring successive steps of denaturation and renaturation of proteins and complicated multistep purification procedures. The novel S-TRAIL fusion protein could successfully overcome all the challenges facing the expression and purification of TRAIL, via a simplified expression system and one-step Ni-affinity chromatography with a yield of up to 8 mg/L of bacterial culture, which is comparable with other studies in this field^35,36^.

### GQD characterization

**Figure 3:**
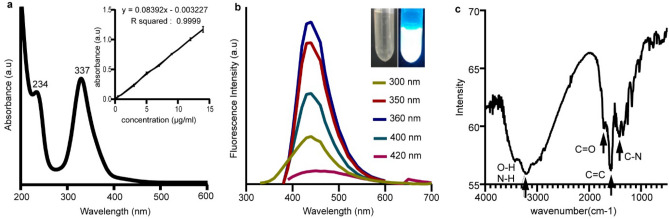
(**a**) UV–Vis absorption spectrum of synthesized GQDs (Inset shows the standard curve drawn based on serial dilution results). (**b**) Photoluminescence spectra of GQDs under different excitation wavelengths of 300–420 nm. The insets are optical images of the solution of GQDs taken under visible light (left) and excited by 360 nm UV light (right). (**c**) FTIR spectrum of GQDs, arrows on the spectrum indicate characteristic bands of functional groups of GQDs.

UV–vis spectroscopy of synthesized GQDs revealed two characteristic absorption bands at 234 and 337 nm (Fig. 3a) related to the $\pi \to \pi^{*}$ and $n \to \pi^{*}$ transitions of C=C and C=O^37,38^. The prepared GQDs exhibited a strong blue emission at 440 nm ($\lambda_{max}$) upon excitation at 300–420 nm; the maximum emission intensity was obtained by excitation at 360 nm (Fig. 3b) This photoluminescence band is excitation-independent, meaning that $\lambda_{max}$ does not shift with the excitation wavelength, which could imply that both the size and the surface hybridization state ($sp^{2}$ clusters) of GQDs are uniform^39^. The FT-IR spectrum of GQDs revealed characteristic stretching bands of O–H and N–H (3000–3500 cm^−1^), vibrational bands of C=O in COOH and CONH (1709, 1667 cm^−1^), and bending vibrations of C=C and C–N (1575–1401 cm^−1^), as shown in Fig. 3c.

### Characterization of S-TRAIL/GQD

**Figure 4:**
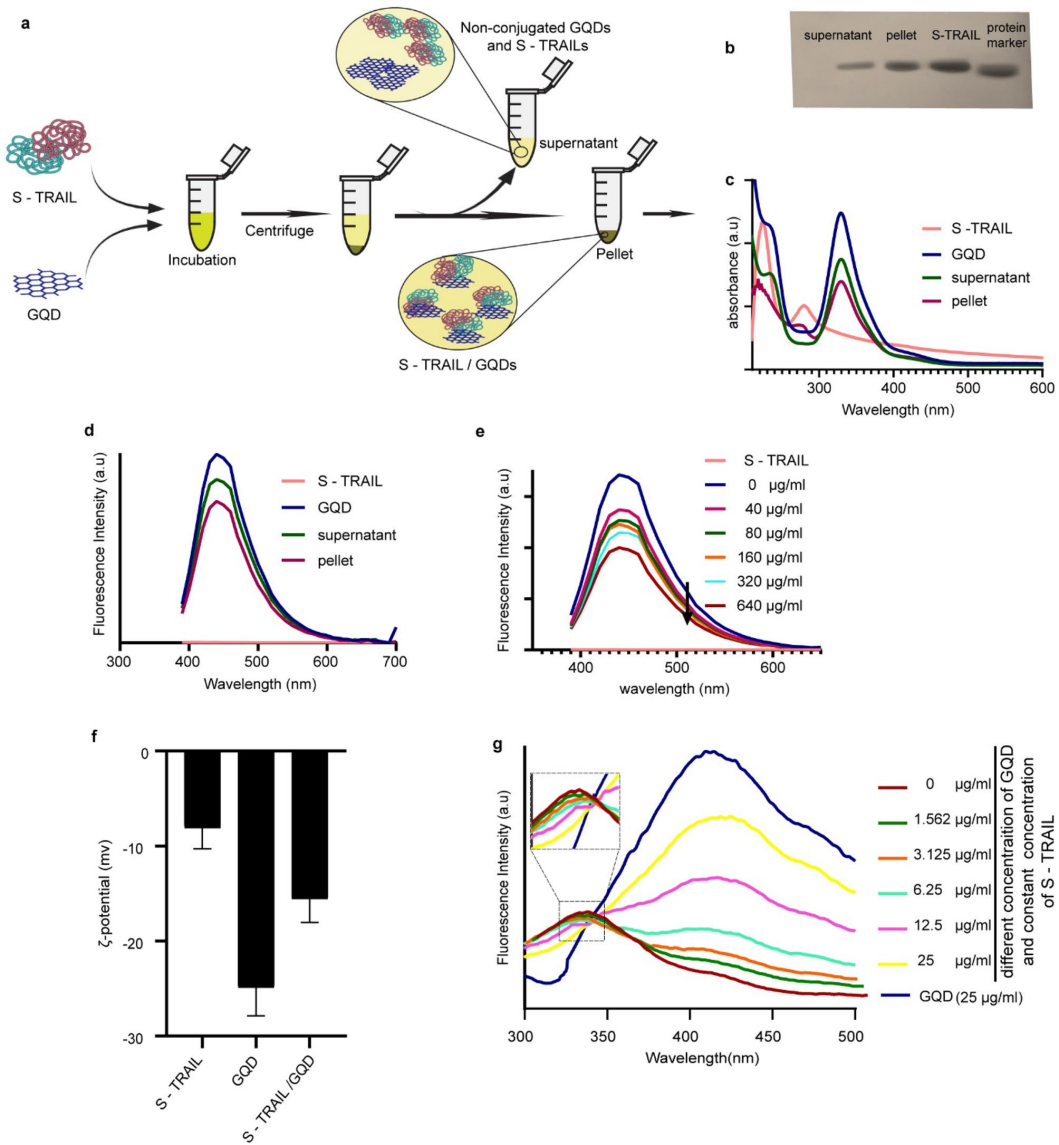
(**a**) Schematic illustration of S-TRAIL/GQD preparation steps. (**b**) Analysis of S-TRAIL content in centrifugation products (both supernatant and pellet) using polyacrylamide gel electrophoresis (12.5%), protein marker is 62KDa. (**c**) UV–visible spectra of S-TRAIL, GQDs and prepared S-TRAIL/GQD after centrifugation. (**d**) Fluorescence spectra of S-TRAIL, GQDs and S-TRAIL/GQD (pellet) and the supernatant upon excitation at 360 nm. (**e**) Fluorescence spectra of GQDs (6 µg/ml) in the presence of different concentrations of S-TRAIL upon excitation at 360 nm. (**f**) ζ-potential of S-TRAIL and GQD before and after conjugation. (**g**) Intrinsic fluorescence of S-TRAIL (160 µg/ml) in the presence of different concentrations of GQD upon excitation at 280 nm.

For S-TRAIL/GQD formation, optimization in terms of concentrations of S-TRAIL and GQDs, incubation time and temperature was done (data not shown). Based on the obtained results, efficient complex formation was accomplished by mixing S-TRAIL (final concentration of 160 µg/ml, pH 7.4) and GQDs (final concentration of 6 µg/ml, pH 7) at the ratio of 1:1 V/V at 25 °C for 30 min under constant gentle shaking in the dark. S-TRAIL/GQDs were then separated by centrifugation from non-conjugated S-TRAILs and GQDs (Fig. 4a). As shown in Fig. 4b, c, the presence of S-TRAIL and GQDs in the pellet was confirmed by SDS-PAGE and UV–Vis spectroscopy. The absorption peaks at 280 nm and 337 nm are related to the S-TRAIL and GQDs, respectively (Fig. 4c). In addition, the fluorescence emission of the pellet (S-TRAIL/GQD) confirmed the presence of GQDs in the precipitated complexes (Fig. 4d). The conjugation of GQDs with S-TRAIL was further investigated by recording the emission spectra of GQDs in the presence of different concentrations of S-TRAIL (Fig. 4e). Concentration-dependent quenching of GQD fluorescence is the result of GQD interactions with S-TRAIL.

By conjugation of GQDs to S-TRAIL, a decrease in ζ-potential from its initial value of -24.9 ± 2.9 mV observed, which could be attributed to electrostatic interactions of hydroxyl and carboxylic acid functional groups of GQDs with positively charged residues of S-layer part of S-TRAIL (Fig. 4f). Changes in intrinsic fluorescence of S-TRAIL in the presence of different concentrations of GQD are also indicative of the presence of interactions between GQDs and S-TRAIL (Fig. 4g).

**Figure 5:**
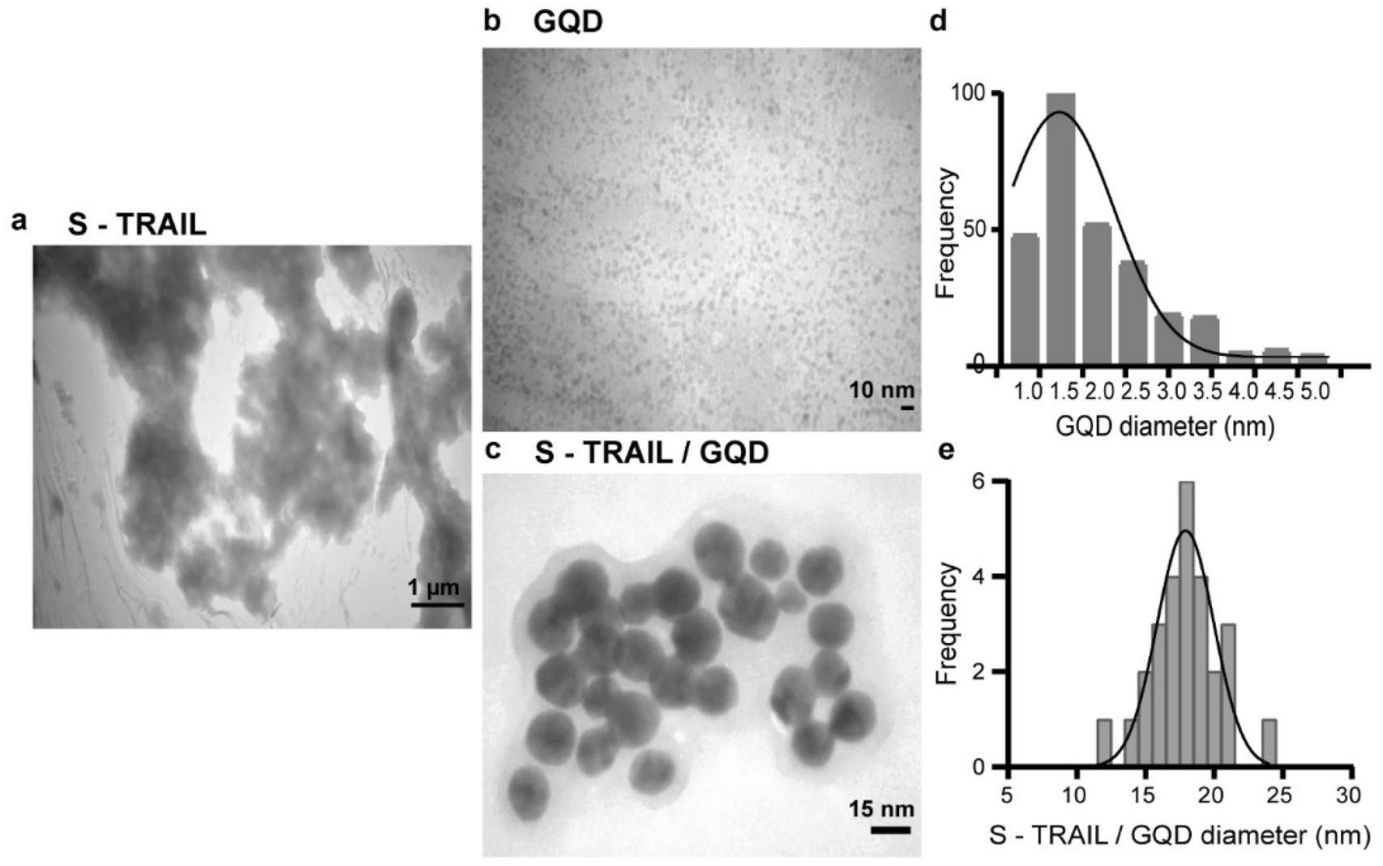
TEM images of (**a**) S-TRAIL, (**b**) GQD and (**c**) S-TRAIL/GQD. (**d**) and (**e**) Size distribution of GQDs and S-TRAIL/GQDs, respectively. Analysis of TEM images was performed by ImageJ software version 1.52.

Furthermore, TEM images of the S-TRAIL, GQD and the S-TRAIL/GQD (Fig. 5) showed that GQDs were in a narrow size distribution with mean diameters of 2.01 ± 0.28 nm (Fig. 5b, d). While the S-TRAIL appeared as amorphous aggregates (Fig. 5a), the S-TRAIL/GQDs were seen as spherical nanoparticles with a mean diameter of 17.77 ± 2.03 nm (Fig. 5c, e). The use of S-TRAIL/GQDs for cancer treatment would take advantage of their preferential accumulation in tumors owing to the enhanced permeability and retention in the tumor site as well as the possibility of tracking and imaging of the delivered system.

### In vitro cytotoxicity and bioactivity assessments

**Figure 6:**
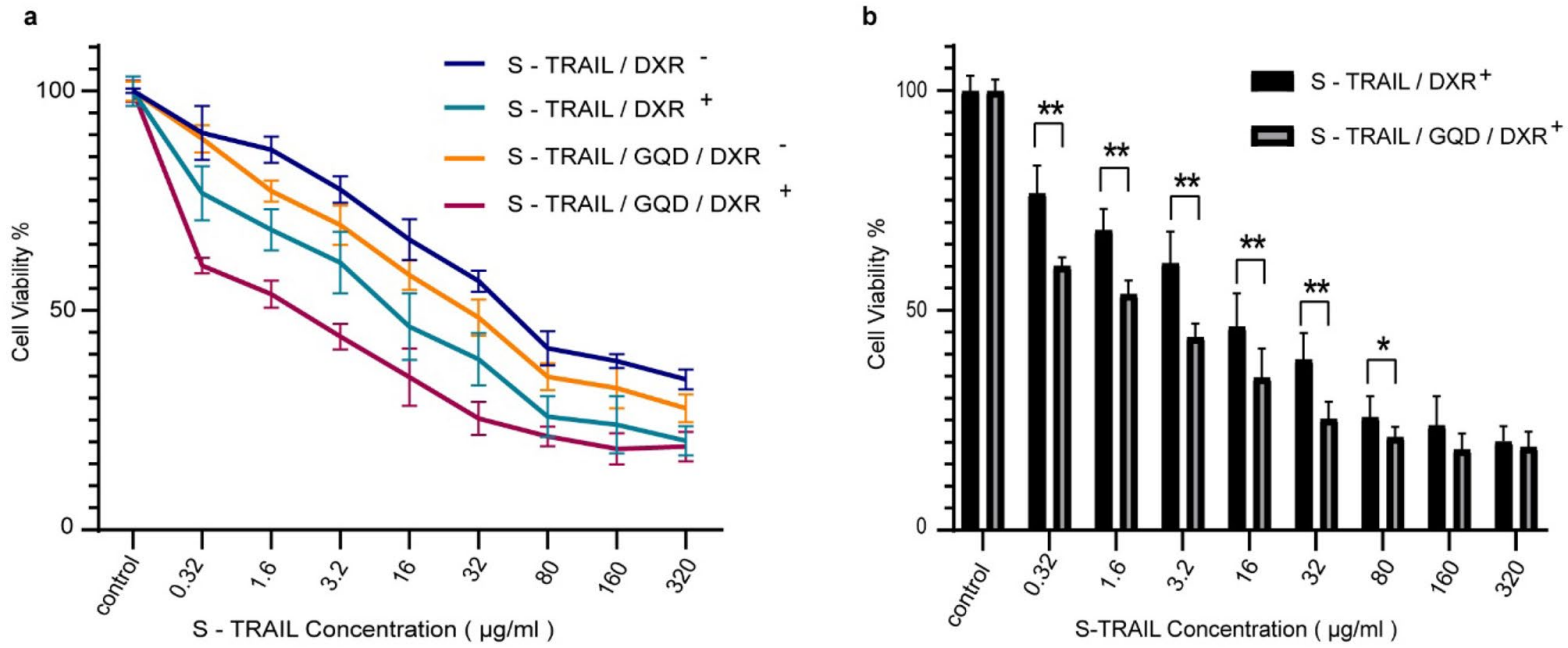
(**a**) In vitro cytotoxic activity of S-TRAIL and S-TRAIL/GQD with/without DXR pretreatment. (**b**) Cytotoxic effects of different concentrations of S-TRAIL and S-TRAIL/GQD following DXR pretreatment for 24 h. The statistical significance was determined using Student’s t-test. $$^{*}p < 0.05,\;^{**}p < 0.01, \;^{***}p < 0.001.$$

In spite of the fact that TRAIL has known as a potent anticancer agent, many cancer cells such as non-small cell lung cancer (NSCLC)^40,41^, prostate cancer^42^, triple-negative breast cancer (TNBC)^43^ and colorectal cancer (CRC)^44,45^ have shown resistance to TRAIL. To address this issue, one proposed strategy was combination therapy (pre-treatment or co-treatment) with chemotherapeutic drugs like cisplatin, doxorubicin (DXR) and 5-fluorouracil to sensitize tumor cells to TRAIL^44,46^. Accordingly, in the present study, HT-29 cells were pre-treated with DXR to sensitize tumor cells and take advantage of TRAIL and DXR synergistic effects mostly through enhancement of pro-caspase-8 activation and mitochondria-dependent death pathway (Bid cleavage)^44,47^ MTT assays were used to evaluate the cytotoxic effects of GQDs, S-layer, S-TRAIL and S-TRAIL/GQD with doxorubicin pretreatment ($DXR^{ + }$) and without doxorubicin pretreatment ($DXR^{ - }$) on the HT-29 human colon carcinoma cell line. GQDs, DXR and S-layer proteins at concentrations that were used for fabrication of the complex had negligible cytotoxic effects on target cells (Fig. S1a–c). It should be mentioned that the S-layer protein concentrations in this experiment were equal to the molar concentration of the S-layer part in the S-TRAIL cytotoxicity assays (Fig. 6a).Concentration-dependent cytotoxic effects of the S-TRAIL and S-TRAIL/GQD on the cells could be clearly seen (Figs. S1d and 6a). The highest tested concentrations of S-TRAIL and S-TRAIL/GQD decreased the viability of DXR-pretreated cells to 20.32 ± 0.02 and 18.99 ± 0.02%, respectively. One plausible explanation of the higher bioactivity of the S-TRAIL/GQD compared to the S-TRAIL, particularly at lower concentrations (Fig. 6a, b), is that GQDs could provide a surface for self-assembly of the S-TRAIL, facilitating the oligomerization of TRIAL monomers. The cytotoxic activity of S-TRAIL and the S-TRAIL/GQD at higher concentrations was not significant, which could be due to the saturation of the cell surface receptors for TRAIL. The results also demonstrated that DXR pretreatment caused a significant increase in cytotoxicity of the S-TRAIL and the S-TRAIL/GQD, which confirmed the undeniable role of DXR pretreatment to overcome therapeutic resistance to TRAIL. HFF-1 cells which have been reported as TRAIL resistant cells^48^ could retain their viability upon S-TRAIL treatment at different concentrations (Fig. S2) and no significant difference in viability of $DXR^{ + }$ and $DXR^{ - }$ cells was seen.

**Figure 7:**
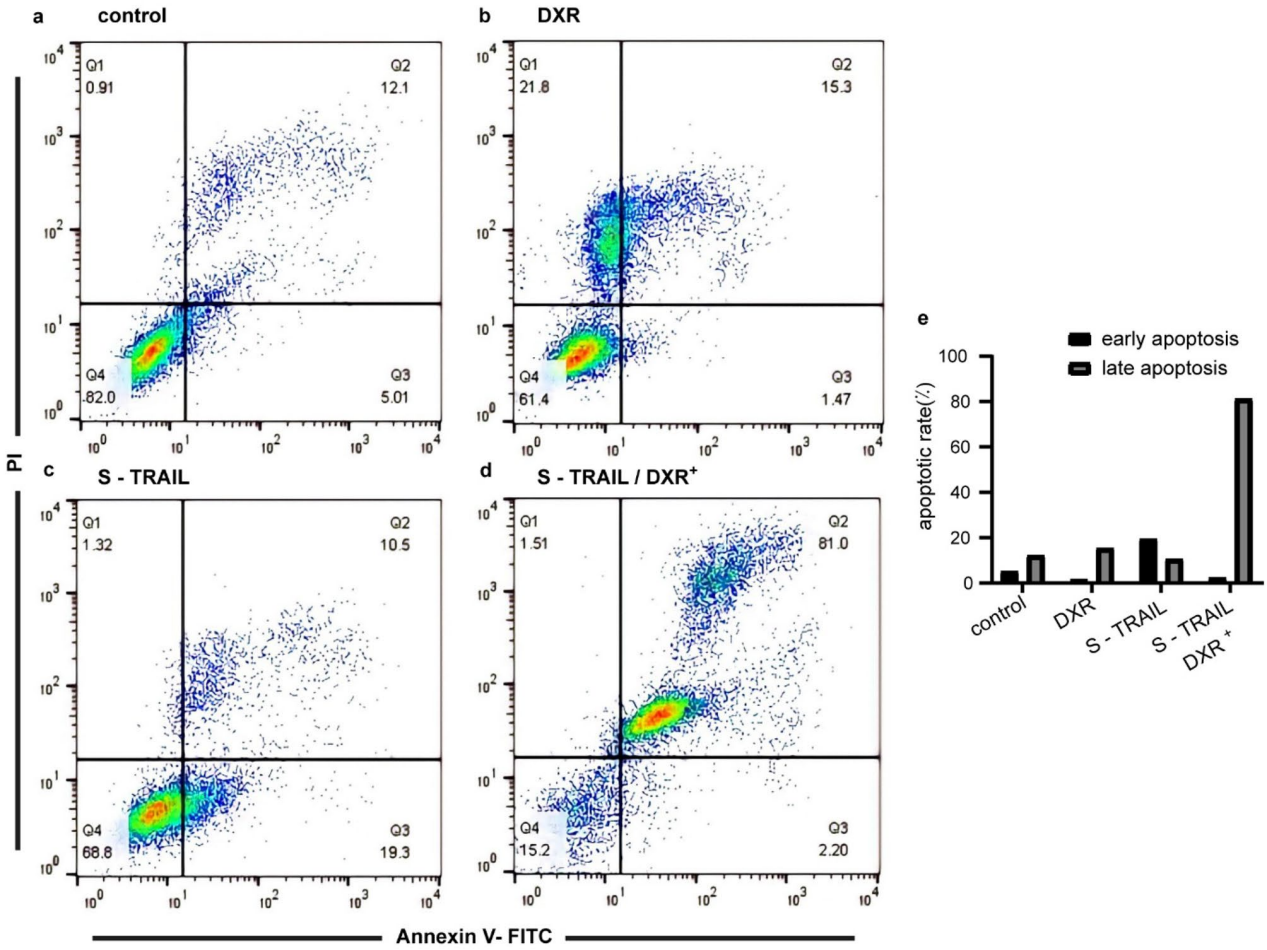
Evaluation of apoptosis induction in HT-29 cells. (**a**) Control cells without any treatment. (**b**) The cells treated with DXR (0.1 µM) for 24 h. (**c**) Treatment with S-TRAIL (160 µg/ml) for 12 h and (**d**) the cells pretreated with DXR (0.1 µM) for 24 h followed by treatment with S-TRAIL (160 µg/ml) for 12 h. (**e**) Quantitative comparison of the apoptosis rate of the cells.

The apoptotic activity of S-TRAIL with/without DXR pretreatment in HT-29 cells were verified by flow cytometry (Annexin V-FITC/PI staining assay)^49^ and compared to untreated cells (Fig. 7a). The cells only treated with doxorubicin showed 16.77% apoptosis (Fig. 7b). Figure 7c clearly demonstrated the apoptotic activity of S-TRAIL. As expected, pretreatment of the cells by DXR significantly increased the population of apoptotic cells from 29.8 to 83.2% of total cells (Fig. 7d, e). The results revealed the potential of sequential therapy with DXR and S-TRAIL to efficiently induce apoptosis in TRAIL-resistant cancer cells.

### S-TRAIL stability study

**Figure 8:**
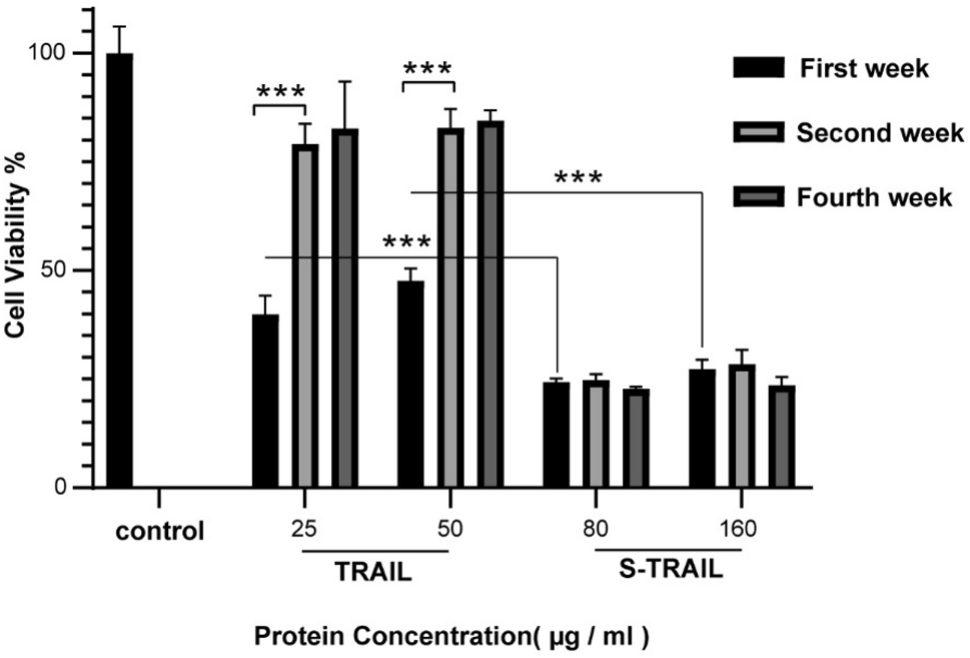
Biological activity of TRAIL and S-TRAIL over 4 weeks storage at 4 °C. The molar concentration of 25 and 50 µg/ml of TRAIL protein are equal to the molar concentration of 80 and 160 µg/ml of S-TRAIL, respectively. Statistical significance was evaluated by repeated ANOVA test and student t-Test. $$^{*}p < 0.05,\;^{**}p < 0.01,\;^{***}p < 0.001$$.

As mentioned earlier, one of the main limitations of TRAIL as a therapeutic agent is its low solubility and stability. Here, the stability of the purified S-TRAIL and TRAIL after 1, 2 and 4 weeks of storage at 4 °C was compared by analyzing their cytotoxic effects on HT-29 cells (Fig. 8). Interestingly, while TRAIL activity was notably diminished after two weeks of storage at 4 °C, the S-TRAIL almost entirely retained its cytotoxic activity even after 4 weeks.

The increased stability and bioactivity of TRAIL by its conjugation to other elements, such as self-assembled albumin nanoparticles^50,51^, hyaluronic acid^5,52^, elastin-like polypeptides (ELPs)^30^ or diphenylalanine (FF)^53^, has been reported earlier. These strategies generally require sophisticated conjugation methods and purification procedures. In this study, the cytotoxic S-TRAIL that was efficiently expressed and purified by a simple method showed enhanced bioactivity and storage stability.

### Fluorescence tracking of the S-TRAIL/GQD complex

**Figure 9:**
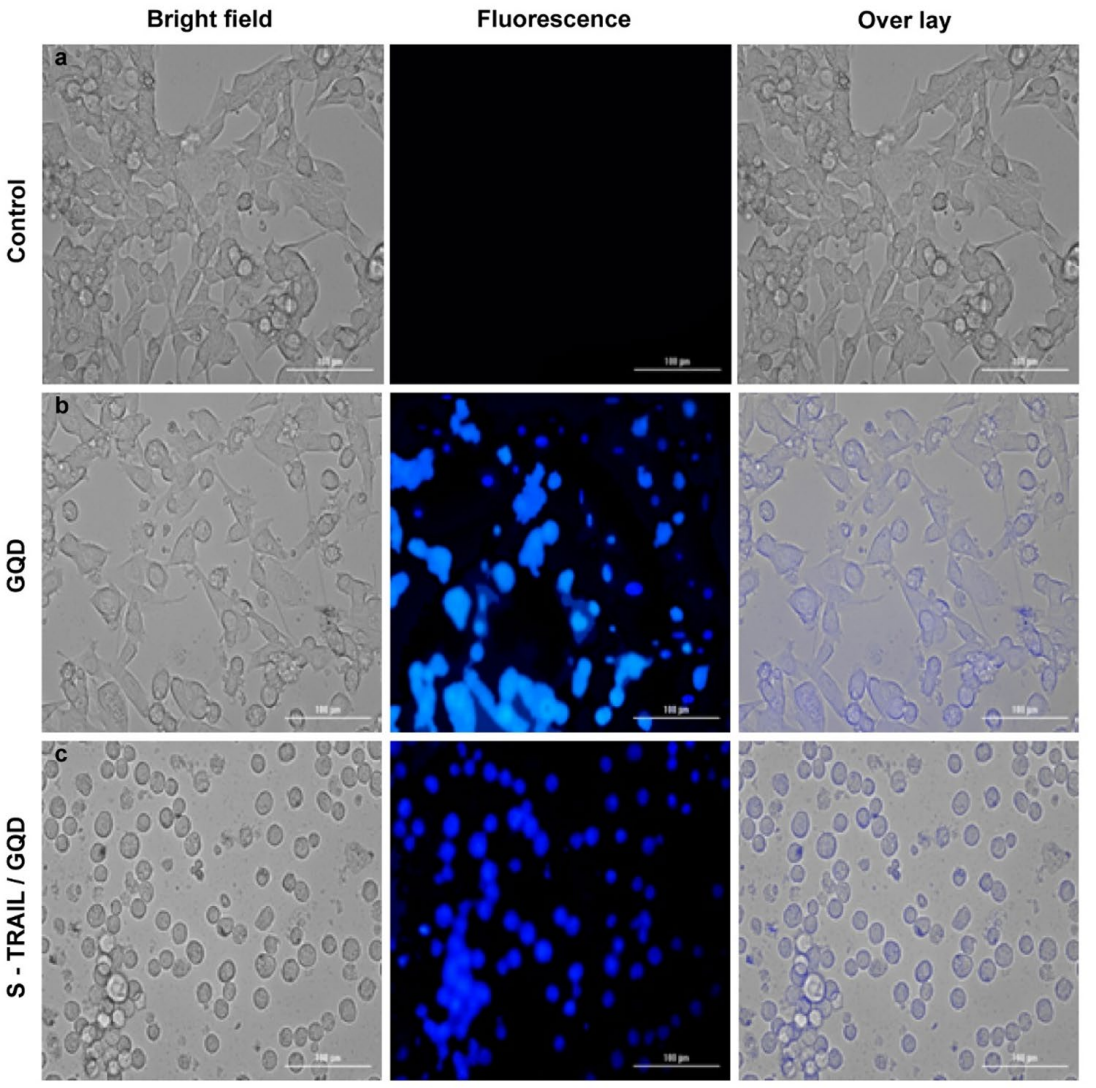
Fluorescence microscopy images of HT-29 cells (**a**) untreated (**b**) treated by GQDs and (**c**) treated by S-TRAIL/GQD for 24 h. In each panel from the left, first image is the bright field, the second image is the fluorescence and the third one is the overlay image of the first and the second images.

Cancer cells treated with the S-TRAIL/GQD were observed by using fluorescence microscopy and compared with control and GQDs treated cells (Fig. 9a–c). TRAIL tracking has been reported earlier by creating fusion proteins of TRAIL and green fluorescent protein^54^ and firefly luciferase^55^. In another study, TRAIL was conjugated with magnetic nanoparticles, which enabled MRI imaging of the treated cells^56^. To the best of our knowledge, this is the first report of tracking TRAIL by taking advantage of GQD fluorescence.

## Conclusion

In this study, a multifunctional nanohybrid system was fabricated by taking advantage of the apoptotic properties of TRAIL, self-assembly of the S-layer proteins and unique optical properties of GQDs. S-TRAIL that was produced by a simple procedure, showed enhanced solubility, stability and bioactivity in comparison with TRAIL. The conjugation of the S-TRAIL with GQDs further enhanced its apoptosis-inducing activity in human colon carcinoma cells. These findings strongly suggest that the S-TRAIL/GQD could be considered as a potent and promising candidate for cancer treatment.

## Methods

### Chemicals, bacterial strains and plasmids

*Escherichia coli* BL21 (DE3) was obtained from Novagen. pET28a expression vector was purchased from Invitrogen. *L. acidophilus* ATCC 4356 was prepared from the microbial collection of Iran and grown in MRS (deMan, Rogosa and Sharpe) medium. Fetal bovine serum (FBS) and Roswell Park Memorial Institute (RPMI 1640) medium were purchased from Gibco. Ni-NTA-Sepharose column obtained from Qiagen. Isopropyl β-d-1-thiogalactopyranoside (IPTG), kanamycin, urea, dialysis bags (12 KDa), monoclonal anti-histidine antibody, doxorubicin, 3-(4,5-s-dimethylthiazol-2-yl)-2,5-diphenyl tetrazolium bromide (MTT) and penicillin/streptomycin were purchased from Sigma–Aldrich. Phenylmethylsulfonyl fluoride (PMSF), citric acid, dithiothreitol (DTT) and ethanol were purchased from Merck. The HT-29 human colon carcinoma cell line was obtained from Pasteur Institute of Iran. The plasmid pET 28a encoding the extracellular domain of human TRAIL (amino acids 114–281) was kindly supplied by Dr S. Hasannia, and it was expressed in SHuffle *E. coli*.

### Preparation and characterization of TRAIL, S-layer and S-TRAIL

#### Construction of S-TRAIL expression vectors

Expression vectors were constructed by inserting the product of SOEing PCR which connected PCR fragments of TRAIL and SlpA, into the vector pET-28a(+).cDNA of extracellular domain of the human TRAIL (residues 114–281) was made from total RNA extracted from human peripheral white blood monocytes. The template for SlpA (residues 1–445) amplification was the genomic DNA extracted from *L. acidophilus.*

TRAIL and SlpA genes were linked using a flexible linker (GGGGSGG). In this regard, four primers, (Table 1) were designed to amplify TRAIL-linker-SlpA. TF and TR were used to amplify the TRAIL-linker and SF and SR were used to amplify the linker-SlpA genes. The PCR products containing TRAIL-linker and linker-SlpA were mixed together for another 15 cycles of PCR without adding any primers. Following construction of TRAIL-linker-SlpA fragment, amplification was carried out using TF and SR primers. TRAIL-linker-SlpA was introduced into the pET-28a(+) at the NheI and SalI restriction sites and *E. coli* BL21(DE3) was transformed with the vectors for the protein expression.

**Table 1:** Sequence of primers required for construction of *trail-linker-SlpA* fragment, cloned in pET-28a (+) plasmid; highlighted part indicates linker sequence.

Primer	Sequence
TF	TAACAGCTAGCATGGTGAGAGAAAGAGGTCCTCAGAGAGTAGCAG
TR	TCCACCTGATCCTCCACCTCCACCAACTAAAAAGGCCCCGAAAAAAC
SF	GGAGGTGGAGGATCAGGTGGAATGAAGAAAAATTTAAGAATCGTTAGC
SR	AATATGTCGACTTATCTAAAGTTTGCAACCTTAACGTAAG

#### Expression and purification of proteins

SHuffle *E. coli* was transformed by a pET28a expression vector containing the extracellular domain of the human TRAIL gene. Both the S-layer and S-TRAIL genes were expressed in *E. coli* BL21 (DE3). Purification of all the mentioned proteins was carried out by Ni-affinity chromatography. Then, purified proteins were dialyzed against dialysis buffers and stored at -20 °C after the addition of 50% glycerol (w/v) until needed. Details have been presented in the supplementary materials. Purified S-layer and S-TRAIL were further analyzed by western blotting using monoclonal anti-His tag antibody and goat anti-mouse AP conjugated antibody.

### Synthesis and characterization of GQDs

N-doped GQDs were prepared using the hydrothermal method by pyrolysis of citric acid and urea according to a reported procedure^37^. Briefly, 0.21 g citric acid and 0.18 g urea were dissolved in 5 ml Milli-Q water. Then, the solution was transferred into a 100 ml Teflon lined stainless steel autoclave and heated at 160 °C in an electric oven for 3 h. After cooling to room temperature, 10 ml ethanol was added to the solution, and centrifugation was performed at 4000 × g for 10 min. The supernatant was dried by a rotary evaporator, and the dried GQDs were dispersed in water and stored in the dark at room temperature until further characterization and use.

Absorption and FTIR spectra of the synthesized GQDs were measured by UV–visible spectroscopy (PerkinElmer, Lambda 25) and FTIR spectroscopy (Tensor 27 FT-IR spectrometer), respectively. Fluorescence spectra were recorded by a Cytation TM 3 multimode detection system (BioTek) at room temperature. The ζ-potential of the GQDs was determined by a dynamic light scattering Zetasizer Nano ZS instrument (Malvern Instruments, UK). TEM images of GQDs were taken on carbon-coated grid Cu Mesh 300 (TEM, Zeiss-EM10C-80 kV).

### Conjugation of S-TRAIL and GQDs

Conjugation of the S-TRAIL and GQDs was accomplished based on adsorption of the oppositely charged S-TRAIL on negatively charged GQDs. To determine the optimum condition of conjugation, different concentrations of S-TRAIL (40, 80, 160, 320 and 640 µg/ml) were mixed with different concentrations of GQDs (3, 6, 12 and 24 µg/ml) at different temperatures (4 °C, 25 °C, 37 °C) and incubation times (15 min to 3 h). To remove non-conjugated S-TRAILs and GQDs, centrifugation at 36,000 g for 30 min at 25 °C (Sigma 3–30 KS centrifuge) was carried out, and the pellet was collected and re dispersed in appropriate buffer.

Conjugation of the S-TRAIL and GQDs was confirmed by polyacrylamide gel electrophoresis (12.5%), UV–visible spectroscopy, intrinsic fluorescence spectroscopy of GQDs and S-TRAIL using a Perkin-Elmer LS-55 fluorescence spectrometer (Perkin-Elmer, USA), ζ-potential measurements and TEM imaging.

### Cytotoxicity assay

HT-29 human colon carcinoma cells and HFF-1 cells were cultured in RPMI 1640 and DMEM, respectively, supplemented with 10% (v/v) fetal bovine serum (FBS) and 1% penicillin/streptomycin in 5% CO2.

Cells were seeded onto 96-well polystyrene culture plates at a density of $1 \times 10^{4}$ cells per well and incubated for 24 h. The cytotoxicity of S-TRAIL and S-TRAIL/GQD at different concentration with doxorubicin pre-treatment ($DXR^{ + }$) and without doxorubicin pretreatment ($DXR^{ - }$) were evaluated using MTT assay. For doxorubicin pretreatment, the cells were pretreated with sub-toxic concentrations of doxorubicin (0.1 µM) for 24 h. Then, the culture media were replaced by fresh RPMI and were subsequently treated with different concentrations of S-TRAIL and S-TRAIL/GQD for 24 h. Then the viability of the cells was evaluated using MTT^57^.

### Flow cytometric analysis of the treated cells

Apoptotic effects of S-TRAIL on the cells with doxorubicin pre-treatment ($DXR^{ + }$) and without doxorubicin pretreatment ($DXR^{ - }$) were assessed by Annexin V-FITC/PI flowcytometry^49^. HT-29 cells were cultured in 12-well tissue culture plates ($8 \times 10^{4} {\text{/well}}$) and allowed to adhere overnight. For doxorubicin pretreatment, the cells were pretreated with sub-toxic concentrations of doxorubicin (0.1 µM) for 24 h. Then, the culture media were replaced by fresh RPMI and were subsequently treated with S-TRAIL for 12 h. The untreated cells and DXR only treated cells were considered as controls. The cells were then stained with Annexin V-FITC/PI and analyzed using a BD FACSCalibur flow cytometer (BD Biosciences, San Jose, CA, USA), with 10,000 events counted per sample. The percentage of apoptotic cells was calculated by FlowJo software.

### S-TRAIL stability study

The cytotoxic activity of TRAIL and S-TRAIL on HT-29 cells was compared at different time intervals after their production using MTT assay. In this regard, HT-29 cells which were pretreated with 0.1 µM DXR were treated by S-TRAIL solution that were kept at 4 °C for 1, 2 and 4 weeks. After incubation for 24 h the cell viability was determined by MTT assay.

### Cell imaging

The cells were exposed to GQD and S-TRAIL/GQDs for 24 h in 6-well cell culture plates ($16 \times 10^{4} {\text{/well}}$) and were then rinsed twice with PBS and imaged by a Cytation TM 3 multimode detection system (BioTek). Doxorubicin pretreatment was performed on cells that were then treated with the S-TRAIL/GQD.

### Statistical analysis

Statistical evaluation of data was performed by unpaired Student's t-test and repeated ANOVA using SPSS version 16 (SPSS Inc, Chicago, IL, USA), and *P* values < 0.05 were considered statistically significant. Data are presented as the mean ± standard deviation (S.D.) D) of at least three different experiments.

### Ethical approval

The experiments with human samples were done according to standard guidelines and approved by the committee for ethics in biomedical research, Tarbiat Modares University.

The authors confirm that informed consent was obtained from the subject whom the blood sample was taken in this research.

## Supplementary Information

Supplementary Information.
