# Supplementary material for: TRAIL/S-layer/graphene quantum dot nanohybrid enhanced stability and anticancer activity of TRAIL on colon cancer cells
Source: Sci Rep. 2022 Apr 7;12:5851. doi: 10.1038/s41598-022-09660-5 (PMC8991220; doi:10.1038/s41598-022-09660-5)
Supplement: Supplementary file 1 — Supplementary Information. [file 41598_2022_9660_MOESM1_ESM.docx]

**Supplementary data**

# Method and Material

## Expression and purification of proteins

### S-layer

E. coli BL21 (DE3) cells were transformed by plasmid pET28a containing Lactobacillus acidophilus S-layer expression gene (encoding residues 1- 445) with poly Histidine tag at both N-terminal and C-terminal. One bacteria colony was inoculated into 10 ml Luria – Bertani (LB) medium supplemented with kanamycin (100 mg/ml ) and grown overnight at 37 °C. Then, 1 ml of pre-culture was added to 250 ml Terrific Broth (TB ) culture media in Hinton flask (Yeast extract 24 g/L, tryptone 20 g/L, Glycerol 4 ml/L , KH_2_PO_4_ 0.017 M, K_2_HPO_4_ 0.072 M ) with 100 mg/ml kanamycin and incubated at 37°C to a density of OD600 = 0.9 , 0.1 mM isopropyl-D-thiogalactopyranoside (IPTG) was then added to induce the expression of the recombinant protein and cultivation was continued for 18 h at 18°C. finally cells were harvested by centrifugation ( 4000 ×g ,15 min, 4°C ), the pellet was re-suspended in lysis buffer (20 mM Tris, 500 mM NaCl, 2 M Urea , 1% Triton x-100, pH 8.0 ) and the lysate was sonicated for 20 cycles (15s on, 45s off, on ice) then the lysate containing S-layers as insoluble aggregates were isolated by centrifugation ( 14000 ×g, 20min, 4°C ) and solubilized in a denaturation buffer containing 20 mM Tris, 500 mM NaCl, 6 M urea, 5mM imidazole, pH 8.0 and incubated at room temperature for 2h followed by centrifugation at 14000 ×g, 20min, 4°C . The supernatant was passed through a Ni-NTA column (Qiagen) which was equilibrated with equilibration buffer (20 mM Tris, 500 mM NaCl, 5 mM imidazole). On column renaturation of S-layers was done by washing the column with buffers containing reducing urea (20 mM Tris, 500 mM NaCl, 8,6,4,2 and 0 M urea, 20mM imidazole, pH 8.0) Afterwards purified S-layers were eluted by elusion buffer (20 mM Tris, 500 mM NaCl, 250 mM Imidazole, pH 8.0 ), dialyzed against dialysis buffer (50 mM Tris, 100 mM NaCl, 2 mM EDTA , pH: 7.4 ) for 24h and kept at -20 °C after addition of 50 % glycerol (w/v).

### TRAIL

SHuffle, an E. coli protein expression strain, which had been transformed by pET28a expression vectors possessing extracellular domain of human TRAIL (amino acids 114-281) with N-terminal histidine tag, incubated in 100 ml LB medium supplemented with kanamycin (100 ${mg.ml}^{-1}$ ) under shaking (220 rpm) at 30 °C .Then, expression was induced using IPTG (0.1 mM, OD600 : 0.6) and incubation followed for 6h. Harvested cells ( centrifugation at 4000 ×g ,15 min, 4°C ), were lysed (50 mM Tris, 500 mM NaCl, 10 mM imidazole, pH 8.0 ) and after sonication (15s on, 45s off, on ice) and centrifugation (14000 ×g, 20min, 4°C ), finally TRAIL was obtained by Ni-NTA column chromatography using wash buffer(50 mM Tris, 500 mM NaCl, 40 mM imidazole, pH 8.0 ) and elusion buffer (50 mM Tris, 500 mM NaCl, 150 mM imidazole, pH 8.0 ). Purified TRAIL was dialyzed against Phosphate buffer saline 1x (PBS ) containing additives to diminish TRAIL aggregation (10% glycerol, 0.08 % sucrose, 0.01 % mannitol , 1mM DTT, 20 µM ZnCl2, pH: 7.4 ) and stored at -20 °C after addition of 50 % glycerol (w/v).

### S – TRAIL

Expression plasmid pET28a possessing polyhistidine tag, human TRAIL gene (encoding residues 114-281), (G4S)3 linker, and Lactobacillus acidophilus S-layer expression gene (encoding residues 1-445) was transformed into component E. coli strain BL21 (DE3). Overnight pre-culture was obtained by inoculating 10 mL of LB medium with one bacterial colony under shaking (180 rpm) at 37 °C, thereafter 1ml of pre-cultured bacteria were cultured in 250 ml Terrific Broth (TB) Medium with 100 ${mg.ml}^{-1}$ kanamycin under shaking (180 rpm) at 37°C until the optical density at 600 nm (OD600) reaches 0.4, then induction the expression of the S – TRAIL was carried out by adding 1 mM of IPTG and cultivation was continued for 5h at 37°C. Finally the cells were harvested (4000 ×g, 15min, 4 °C) and re-suspended in 10ml of lysis buffer (20 mM Tris, 500 mM NaCl, 5 mM imidazole, 1% Triton x-100, 0.1mM PMSF, pH 11.0). The cells were disrupted using 15 min sonication on ice (20s pulse, 40 s pause). Supernatant was collected by centrifugation (14000 ×g, 15min, 4°C ) for puriﬁcation process. The supernatant was loaded onto Ni-NTA (nitrilotriacetic acid) sepharose column, equilibrated with equilibration buffer (20 mM Tris, 500 mM NaCl, 5 mM imidazole). The column was washed by stepwise wash buffers containing 20 mM Tris, 1M NaCl, 20, 40 and 60 mM Imidazole, pH 8, then the attached S – TRAIL were eluted with elusion buffer (20 mM Tris, 500 mM NaCl, 250 mM Imidazole, pH 8), dialyzed against dialysis buffer (50 mM Tris, 100 mM NaCl, 2 mM EDTA, pH 7.4) and stored at -20 °C after addition of 50 % glycerol (w/v) until needed. The purified protein were analysed by Western blotting using monoclonal Anti-His tag antibody and goat anti-mouse AP conjugate antibody.

# Result

## In vitro cytotoxicity and bioactivity of GQD, Doxorubicin and S-layer protein

HT-29 cells were treated by different concentration of doxorubicin (DXR) for 24 and 48h (Fig. S1a). DXR at concentration of 0.1 µg/ml was used for pretreatment of the cells followed by treatment by S-TRAIL and S-TRAIL/GQDs. DXR was reported to sensitize cancer cells to TRAIL through increasing recruitment of FADD and pro-caspase-8. [^1^](#lacour1)


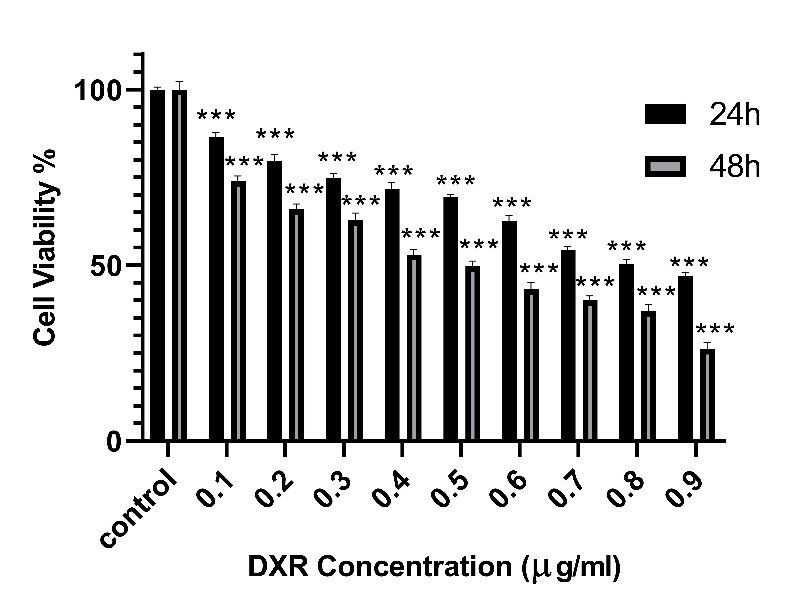

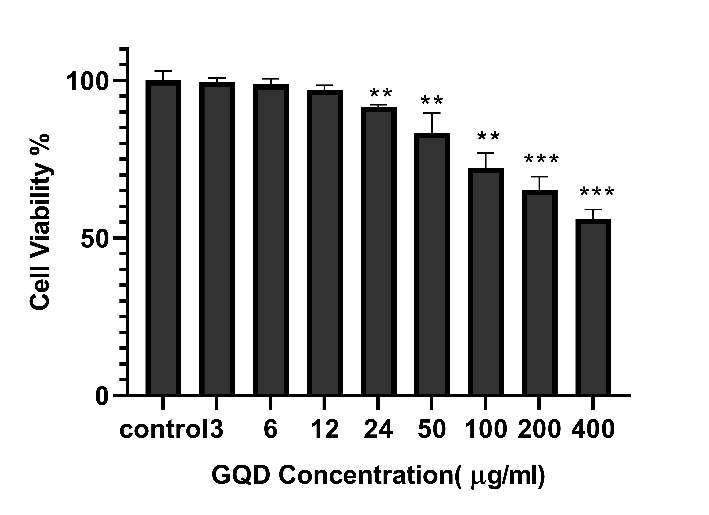


**a**

**b**


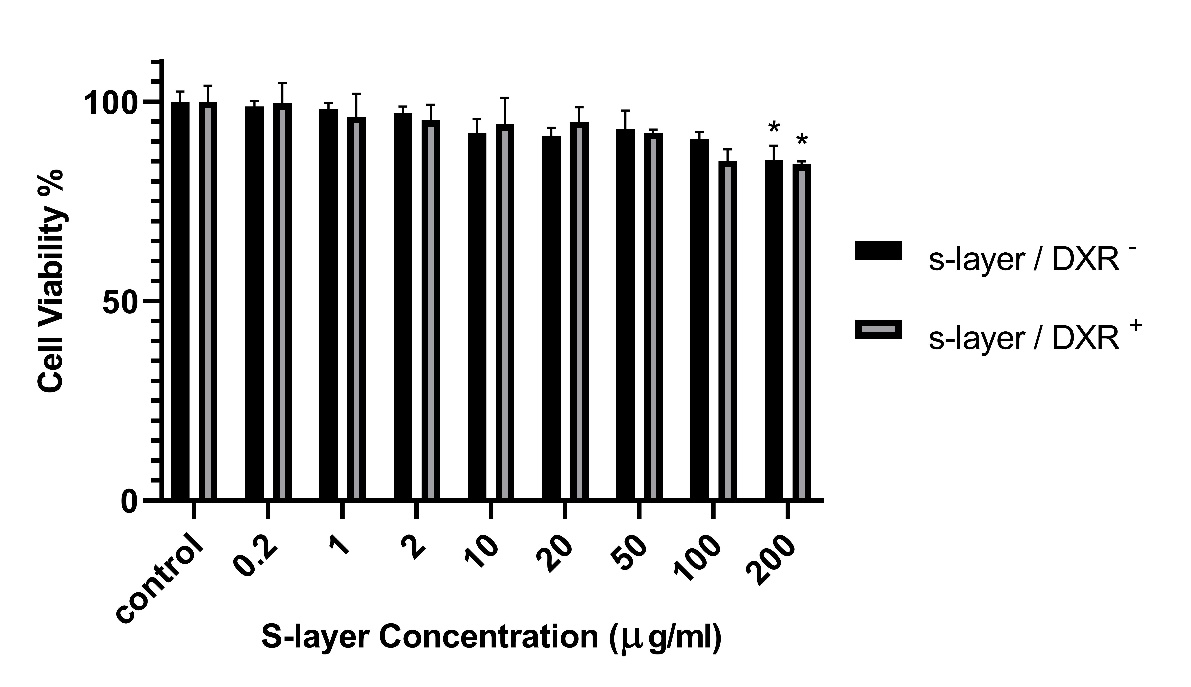


**c**

**d**


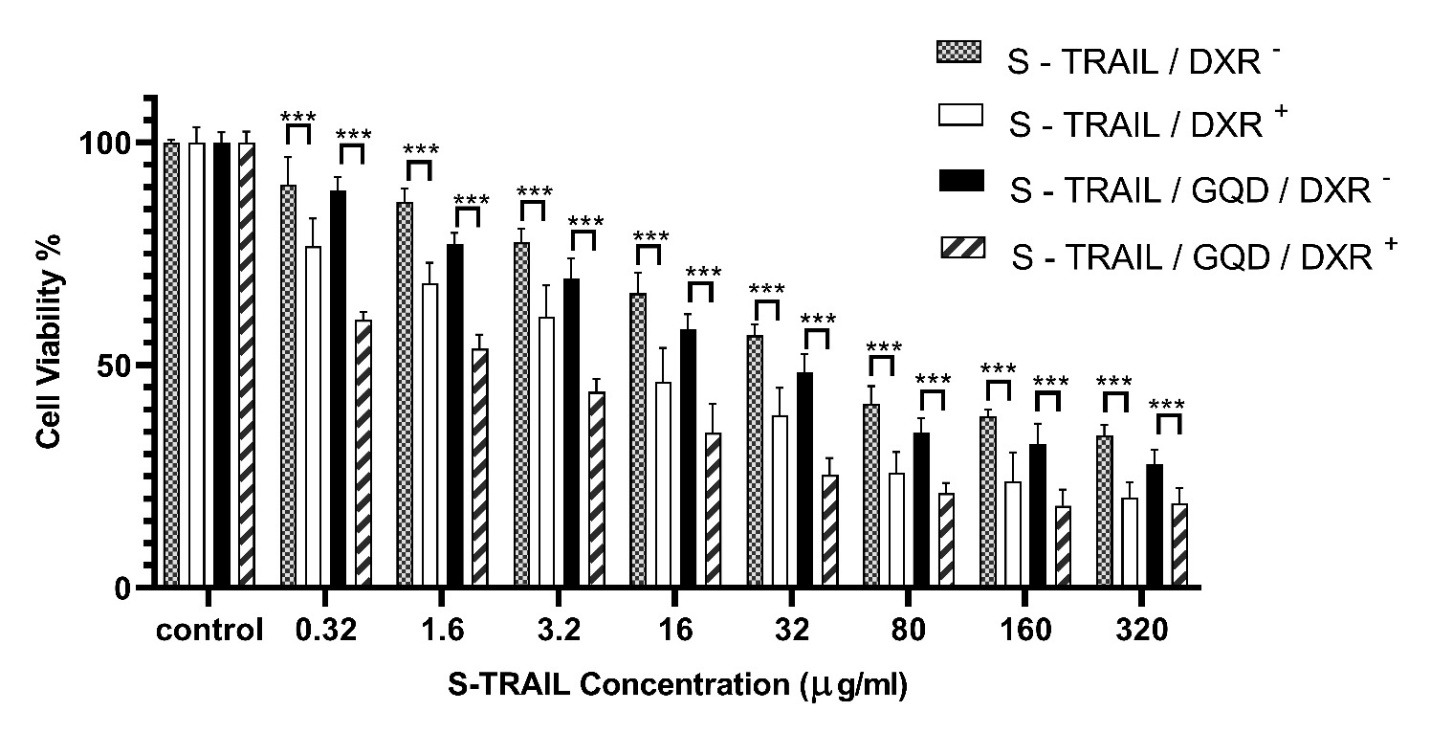


d

Figure S1. In vitro cytotoxicity of a) different doxorubicin (DRX) concentration after 24 and 48h incubation b) different concentration of GQDs after 24h incubation and c) different concentration of S-layer protein after 24h incubation with HT-29 cells . Cells without any treatment served as control cells d) cytotoxicity comparison of S – TRAIL and S – TRAIL / GQD with and without pretreatment with DRX. $*p<0.05, **p<0.01, ***p <0.001$


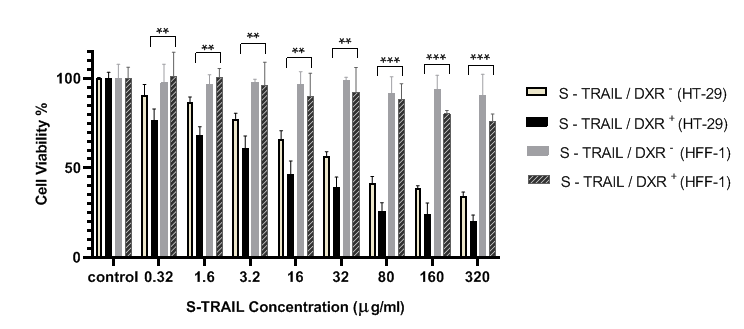


Figure S2. In vitro cytotoxicity of S – TRAIL with and without pretreatment with DRX on HT-29 and HFF-1 cells. The statistical significance was determined using Student’s t-test. $*p<0.05, **p<0.01, ***p <0.001$

Original gel/blot figures


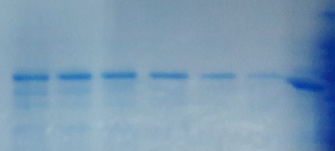


Figure .2a

Figure .4b


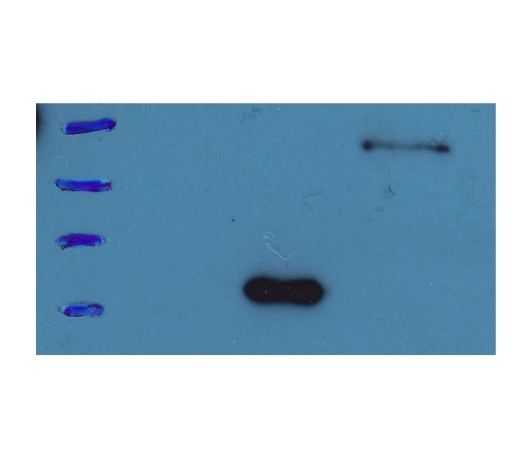

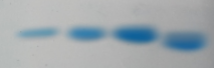


Figure .2b

**References**

[1. Lacour, S., Micheau, O., Hammann, A., Oncogene, V. D.- & 2003, undefined. Chemotherapy enhances TNF-related apoptosis-inducing ligand DISC assembly in HT29 human colon cancer cells.](#lacour) *[nature.com](#lacour)*[.](#lacour)
